# Fluorescent Molecules That Help Reveal Previously Unidentified Neural Connections in Adult, Neonatal and Peripubertal Mammals

**DOI:** 10.3390/ijms241914478

**Published:** 2023-09-23

**Authors:** Enikő Vasziné Szabó, Katalin Köves, Ágnes Csáki

**Affiliations:** 1 Department of Restorative Dentistry and Endodontics, Faculty of Dentistry, Semmelweis University, Szentkirályi u. 47, H-1088 Budapest, Hungary; 2 Department of Anatomy, Histology and Embryology, Faculty of Medicine, Semmelweis University, Tűzoltó u. 58, H-1094 Budapest, Hungary; koves.katalin@semmelweis.hu (K.K.); csaki.agnes@semmelweis.hu (Á.C.)

**Keywords:** neuronal pathways, fluorescent tracers, autonomic innervation of gingiva and lip, genetically modified mice, centrifugal visual fibers, pineal body

## Abstract

One hundred and twenty-five years ago there was a lively discussion between Hungarian and Spanish neuroscientists on the nature of neural connections. The question was whether the neurofibrils run from one neuron to the next and connect neurons as a continuous network or the fibrils form an internal skeleton in the neurons and do not leave the cell; however, there is close contact between the neurons. About 50 years later, the invention of the electron microscope solved the problem. Close contacts between individual neurons were identified and named as synapses. In the following years, the need arose to explore distant connections between neuronal structures. Tracing techniques entered neuroscience. There are three major groups of tracers: (A) non-transsynaptic tracers used to find direct connections between two neuronal structures; (B) tracers passing gap junctions; (C) transsynaptic tracers passing synapses that are suitable to explore multineuronal circuits. According to the direction of the transport mechanism, the tracer may be ante- or retrograde. In this review, we focus on the ever-increasing number of fluorescent tracers that we have also used in our studies. The advantage of the use of these molecules is that the fluorescence of the tracer can be seen in histological sections without any other processes. Genes encoding fluorescent molecules can be inserted in various neuropeptide or neurotransmitter expressing transcriptomes. This makes it possible to study the anatomy, development or functional relations of these neuronal networks in transgenic animals.

## 1. Introduction

The aim of this work is to review the discovery of connections between neurons (Section 2) and to review the types of tracers that can be used to examine neuronal circuits (Section 3). First, we focus on the fluorescent non-transsynaptic tracers because they provide the possibility to demonstrate their presence using fluorescence microscopy without any histochemical or immunohistochemical procedures (Section 4). Then we review the transsynaptic virus tracers (Section 5) and the discovery of green and other fluorescent proteins, the genes of which can be inserted into the viral genome. Later, fluorescently labeled viruses were widely used to explore neuronal connections (Section 6). We shortly discuss the role of fluorescent marker proteins, the genes of which can be inserted in various neuropeptide, neurotransmitter or other specific cell line genomes. In these so-called knock-in animals, specific systems can be studied with a fluorescent microscope without any previous procedure (Section 7). In the next part of paper, we gathered the different mechanisms of how tracers can enter the neurons, and what phenomenon determines the direction of spreading along the neurons (Section 8). After that we present our own experiments carried out in adult, neonatal and peripubertal animals using fluorescent protein-labeled virus tracers (Section 9). Finally, we summarize the most important advances and future opportunities (Section 10).

## 2. A Brief History of the Discovery of Neuronal Connections (Contiguity vs. Continuity)

More than 100 years ago, it was assumed by Apáthy [1] and Bethe [2] that the nerve impulses move along neurofibrils. According to their hypothesis, the neurofibrils run from one neuron to another continuously and connect neurons as a continuous network. This theory was called “continuity”. At the same time, Ramon y Cajal, a Spanish neuroscientist [3], realized that the fibrils form an internal skeleton in the neurons, which do not leave the cell; however, there exists a close connection between the neurons. This connection was named “synapse”. This theory was called “contiguity”. Ramon y Cajal received a Nobel Prize for this discovery. After the invention of the electron microscope (EM) by Ruska and Knoll [4], Cajal’s theory was confirmed. With the use of transmission electron microscopical investigation (TEM), the structure of synapses was described in detail by Luse [5]. She stated, “At the present time the significance of these neural interrelationships is problematical”. Before knowing the morphology of synapses, Otto Loewi [6] discovered that there is a chemical transmission between neurons. Loewi with Henry Dale also received the Nobel Prize. Despite Luse’s doubts, her results provided a morphological basis for the chemical transmission. Later, when the connections between nerve elements were further characterized, a new type of connection was explored. It was an electrical synapse called a gap junction. Gap junctions are present not only between nerve elements, but also between other cell types [7].

In the following years, the need arose to explore neural connections. Tracing techniques entered neuroscience. There are several important criteria that were defined by Saleeba and her co-workers [8]:

(1) The tracer should not influence the transport system of the neuron.
(2) The tracer should be minimally neurotoxic.
(3) The tracer should be present in large enough concentrations for identification.

## 3. Classification of Neuronal Tracers

A useful method is the use of tracers for the exploration of neuronal connections. Tracers are molecules that can be taken up by neurons and transported in either ante- or retrograde directions. Several types of tracers are available.

(A) Non-transsynaptic tracers are used to find direct one-neuronal connections between two neuronal structures. These tracers do not pass chemical or electrical synapses and cannot be picked up from the blood.

1. At the beginning, tritiated amino acids (leucin or proline) were used to explore the terminal field of neuronal connections. These tracers were injected into a given area of the brain and then they were taken up by the injured neuronal cell bodies and transported along the axons. Autoradiography was used to visualize the transported amino acids. This method was introduced in the 1960s [9].
2. Later, many other tracers were introduced which meet the above-mentioned criteria. The number of molecules is abundant (plant lectins: wheat germ agglutinin (WGA) and phaseolus vulgaris leucoagglutinin (PHA-L), dextran amine DAs, choleratoxin, etc.). In this review, we focus on the ever-increasing number of fluorescent tracers.
3. Some tracers can be picked up from the blood, where the blood brain barrier is missing (circum ventricular organs: median eminence, area postrema, neurohypophysis, organum vasculosum of the lamina terminalis, pineal body, subcommissural organ, and subfornical organ), but do not pass through synapses. These tracers may be administered intravenously, intraperitoneally or somewhere peripherally as well. For example, intraperitoneal injection of FluoroGold (FG) was able to label hypophyseotropic neurons in the hypothalamus [10,11].

(B) Some tracers are able to pass gap junctions (electrical synapsis). Mills and Massey [12] characterized the gap junctions in the retina using several types of tracers constructed from neurobiotin (biotin ethylenediamine). These tracers had low molecular weight (MW) of 244–555 kDa; therefore, they were suitable to demonstrate the diversity of electrical synapses. Other small molecules, such as lucifer yellow [13] and biotinylated dextran amine (BDA) with 3000 or lower MW, were also shown to pass gap junctions. Trementozzi and co-workers [14] investigated the transport of dextran chains through gap junctions in the range of 10–70 kDa. It was found that not the free dextran but dextran chains of up to 40 kDa can diffuse through at least five cell layers in a gap junction-dependent manner within 30 min in cell cultures.
(C) Transsynaptic tracers can pass synapses. These are the neurotropic viruses. When a virus enters a neuron, it replicates and then it enters the next member of the neuronal chain and replicates again [15]. Finally, the virus infection becomes widespread and the animal dies. It is important to choose a suitable survival time for the infected animal.

## 4. Fluorescent Molecules as Non-Transsynaptic Tracers

Fluorescent dyes make the visualization of neuronal connections possible without histochemical processing. Fluorescent organic dye was first introduced by Kristensson in 1970 [16] by injecting Evans Blue (EB) into the gastrocnemius muscle of suckling mice. As a result of retrograde transport of the fluorochrome, the corresponding motor neurons of the spinal cord were visualized. Limitations, associated with the use of such dyes, are the following: the rapid bleaching, the uptake of these fluorochromes by fibers of passage, their limited cellular definition and the diffusion of the dye from the labeled neurons [17]. EB is a retrograde fluorescent dye which was first used by Herbert McLean Evans in 1914 for diagnostic purposes [18]. It binds strongly to serum albumin, thereby becoming a protein tracer [19]. EB is able to emit red fluorescence when excited with green light [20]. EB was coupled with bovine serum albumin for the tracing of neuronal synapses and was made visible using fluorescence microscopy. It was first used for multiple fluorescent tracing by Kristensson [16] and by Steward and Scoville [21]. Another fluorescent tracer, a mixture of 4′-6-diamidino-2-phenylin-dol-2-HCl (DAPI) and primuline (DAPI-Pr), was successfully used by van der Kooy and co-workers [22] to visualize divergent axon collaterals that fluoresced in blue. Later, in 1980, new retrograde fluorescent tracers were introduced, nuclear yellow (NY) and bisbenzimide (Bb), which primarily label neuronal nucleus and fast blue (FB) and true blue (TB) that label neuronal cytoplasm in blue [23,24]. All dyes were excited at the same wavelength of 360 nm. These dyes are able to be transported over long distances. With these fluorochromes, the double labeling of axon collaterals was made possible. NY is transported quite rapidly along the axon and it has a short survival time. If the animal lives longer, the dye migrates out of the labeled neuron resulting in false labeling [23]. FB and TB are transported much slower and stay in the labeled neuron for a longer time than NY. For successful double labeling, it is important to know the survival time of the dyes and to determine the injection time of each tracer. A new fluorescent compound, diamidino yellow dihydrochloride (DY), was described and used first by Keizer and co-workers [25] to overcome this problem. Combining DY and TB or FB allowed the double labeling of neurons to be possible. When NY and Bb are present together, a disadvantage is that they are also transported anterogradely through the axons; however, this anterograde transport does not produce fluorescent labeling of the axons [17]. A new fluorescent tracer, a stilbene compound, 4-acetamido-4′-isothiocyanostilbene-2,2′-disulfonic acid (SITS) was introduced in 1982. It is only taken up by axon terminals and can be used as a purely retrograde tracer. It produces yellow fluorescence in the cytoplasm and resists fading. It will not diffuse from the neuron even after a long survival time [26]. Its disadvantage is that its commercial preparations have an impurity interfering with labeling. Other stilbene compounds, such as Stilbene Gold (SG) and FG, were introduced to overcome this disadvantage of SITS [27]). SG and FG also have a long survival time and can easily be combined with other histochemical techniques and simultaneously used with other retrograde tracer molecules. A completely new method for fluorescent tracing was proposed by the development of fluorescent latex microspheres with a diameter of 0.02–0.2 µm. These beads are easily transported by neurons and can label cell cytoplasm. Their survival time is 24 h to one month. They are resistant to fading and can be used in the double labeling technique using latex spheres with two different fluorescences. Rhodamine-labeled fluorescent latex microspheres show red, while green fluorescent latex microspheres show green fluorescence [28].

## 5. Transsynaptic Tracers

Nowadays, three well-defined virus strains are usually used as transsynaptic tracers: pseudorabies virus (PRV), herpes simplex virus (HSV) and vesicular stomatitis virus (VSV) [29]).

### 5.1. Attenuated Pseudorabies Virus Bartha (PRV-Ba)

This virus was first developed as a vaccine [30]. This virus cannot infect higher primates [31]. At the beginning, antibody against the virus protein was used to visualize the virus location in the nervous system. Later, Mettenleiter and Rauh [32] inserted a gX-β galactosidase fusion gene (LacZ gene) in the PRV genome, replacing a non-essential genomic region. This mutant PRV is often used to investigate neuronal pathways. The virus can be revealed by β-galactosidase antibody. The virus researchers discovered that gE and gI genes are responsible for enhancing the virulence of viruses. Interestingly, removing these genes also eliminates the anterograde spread of the virus. After removal of the gE and gI genes, viruses can only be transported retrogradely [33,34].

### 5.2. Herpes Simplex Virus (HSV)

Ho and Mocarski [35] inserted a modified Escherichia coli LacZ gene into the HSV-1 genome, disrupting the viral thymidine kinase gene. The LacZ gene product is β-galactosidase. The presence of this virus can also be made visible by β-galactosidase antibody. This modified virus shows mild cytotoxicity, and its spread is not affected by the manipulation.

### 5.3. Vesicular Stomatitis Virus (VSV)

VSV was also introduced in research as neurotropic transsynaptic virus. Beier and co-workers [36] created the VSV vector which encodes a glycoprotein from the lymphocytic choriomeningitis virus.

## 6. Discovery of Green Fluorescent Protein (GFP) and Other Fluorescent Proteins

Osamu Shimomura, a Japanese marine biologist who received a Nobel Prize in 2008 [37], isolated a protein from a jellyfish. This protein emitted blue light [38]. The blue light of the protein was absorbed by a second jellyfish protein, which in turn re-emitted green light. It was introduced in the literature as GFP [39]. GFP is stable, easy to recognize with fluorescence microscopy [40]. The modification of GFP resulted in different mutants with red fluorescence excitation [41]. Tsien and collaborators [42] modified the structure of GFP. This modification produced a new protein that shone very brightly and produced different colors: cyan, blue and yellow. The fusion of mCherry and GFP resulted in a photo-resistant product [43]. Matz and his co-workers [44] isolated six GFP-like proteins from fluorescent discoma corals (Ds). One was red and was later named DsRED. Merzlyak and her co-workers [45] described a Tag red fluorescent protein (TagRFP), which was bright, exhibited prolonged fluorescence lifetime and high pH stability.

Labeling neurotropic viruses with fluorescent molecules was a milestone in the study of neuronal circuits. Jöns and Mettenleiter [46] used attenuated PRV strain Bartha in their experiments. They inserted GFP RNA in the non-essential glycoprotein G (gG) gene. This virus emitted green fluorescence. With the use of GFP or other fluorescent protein-labeled viruses, many advances were made in exploring neuronal circuits. Boldogkői and his co-workers produced PRV viruses which are not so toxic to experimental animals, even at the late stages of infection. They stated: “These viruses are suitable to give information not only about the early, but about the late phase of infection. They are called timer PRVs [47]. If two retrograde viruses express two different proteins (GFP and DsRED with different kinetics and intracellular distribution, one (membrane-targeted green fluorescence) appears at the early stage of infection (primary fluorescent protein) while the other (soluble red reporter) is detectable several hours later (secondary fluorescent protein)”.

## 7. Fluorescent Protein Expressing Transgenic Animals in the Research of Neuronal Networks

The creation of transgenic knock-in mice was a significant advance in the study of neurotransmitter and neuropeptide systems. GFP or other fluorescent protein genes were inserted in the neurotransmitter or neuropeptide or other specific cell line genome, allowing the visualization of the anatomy, development and connections of these neurons or their answer to various experimental manipulations. The expression of GFP persists during the lifetime of these animals and also appears in the offspring [48].

Many types of transgenic mice are now available. CRFp3.0CreGFP transgenic mice, expressing GFP in the corticotropic hormone-releasing factor (CRF) synthesizing cells, were generated by Martin and co-workers [49]. These specific CRF cells exhibited green fluorescence and CRF immunohistochemistry confirmed their CRF nature [50]. Transgenic mice with GFP expressing vasoactive intestinal polypeptide (VIP) neurons were generated at Rockefeller University. These so called “knock-in” mice were successfully used to investigate the VIP neuronal system in combination with the tract tracing technique and immunohistochemistry. Prönneke and her co-workers [51] characterized gamma aminobutiric acid (GABA) neurons in the cerebral cortex using VIPcre/tdTomato knock-in mice and they revealed layer-specific differences. GAD (glutamate decarboxylase) 67-GFP knock-in transgenic mice are also available [52]. Xu and co-workers [53] characterized the GABAerg inhibitory neurons in glutamate decarboxylase 67 KDa isoform (GAD67)-GFP knock-in transgenic mice using parvalbumin (PV), somatostatin (SOM), calretinin (CR) and cholecystokinin (CCK) immunohistochemistry. Chi-Sung Chiu and his co-workers [54] constructed a strain of knock-in mice that had alpha-1,3-mannosyl-glycoprotein 2-beta-N-acetylglucosaminyl-transferase (mGAT1)-FP fusion gene in place of the wild-type GAT1 gene. The pattern of fluorescence in brain slices agreed with previous immunocytochemical observations. Thy1-YFP-H transgenic knock-in mice express yellow fluorescence in cell surface glycoprotein at high levels in motor and sensory neurons, as well as in subsets of central neurons. Yellow fluorescent protein (YFP) is a strong and specific “Golgi-like” vital marker. These knock-in mice were successfully used to map neurons and axon pathways in adult and developing mice [55,56]. Many other knock-in mice having gonadotropic hormone-releasing hormone (GnRH)-GFP [57], melanocortin-4 receptor (MC4-R-GFP) [58], nestin-GFP [59], calbindin (Calb)-GFP [60] or choline acethyltransferase (ChAT)eGFP cell lines are also available [61].

## 8. The Mechanism of Action of Tracers

The question arises how the tracers can enter the neurons. Several types of mechanisms have been identified for how the tracers pass the cell membrane of neurons (surveyed by Saleeba et al. [8]):

1. Weak alkaline tracers may cross the cell membrane and may be trapped by lysosomes and endosomes which are acid cellular components.
2. Tracers may be taken up by non-specific passive endocytosis.
3. Tracers may bind to the neuron by sugar-mediated uptake.
4. Other tracers enter the neurons at the terminals and are carried by vesicles to the cell body.
5. Latex microspheres can enter the damaged membrane surface, but there is a minimal entry through undamaged fiber membrane surfaces as well.
6. Several tracers enter injured neurons at the injection site and spread bidirectionally with diffusion.
7. Static viral tracers enter the neuron via interaction between glucans or protein receptors expressed on the cell membrane and components of a viral vector. If the receptor is present on the terminals, it results in retrograde spreading. If the receptor is present on the soma, it results in anterograde spreading. Researchers can manipulate the direction of spread.

## 9. Applications of GFP-Labeled Virus Tracer in Our Study in Adult Rats

### 9.1. Autonomic Innervation of Mandibular Gingiva and Lower Lip

Fluorescent-labeled virus was used to define central command neurons in the brain. It is well established that both sympathetic and parasympathetic premotor neurons reside in the brainstem and the hypothalamus and give rise to the descending autonomic pathways [62]. In our experiment, the GFP-labeled retrograde-spreading virus (MemGreenPRV-R, generated by Boldogkői et al. [34,47]) was injected into the gingiva or the lower lip of intact rats. Four days later, the virus injection resulted in labeling of the post- and preganglionic structures including the three cervical ganglia and the intermediolateral cell column of the spinal cord (IML). Premotor neurons in the brainstem and hypothalamus were found. Labeled neurons were located in the rostral ventrolateral medulla (rVLM), gigantocellular reticula neurons (Gig), locus ceruleus (LC), paraventricular nucleus (PVN) and perifornical area (Pf). Sympathectomy prevented the labeling in the cervical ganglia, rVLM and LC; however, it persisted in the rest of the labeled structures (PVN, Pf and Gig). This means that these latter neurons are the third-order parasympathetic neurons [63]. Double labeling (MemGreenPRV-R inoculation and oxytocin immunohistochemistry) showed that some hypothalamic oxytocinergic premotor neurons innervated preganglionic neurons in the spinal cord. We found that parasympathetic postganglionic neurons related to the lower gingiva are present in the otic and those related to the lower lip are present in the otic and submandibular ganglia and the preganglionic neurons are in the salivatory nuclei. As mentioned above, sympathectomy revealed that the third-order parasympathetic neurons were found in the Gig, PVN and Pf [63]. In both intact and sympathectomized rats, the virus-labeled neurons of the PVN showed oxytocin and vasopressin, but not CCK and CRF immunoreactivities. In the Pf, the virus-labeled neurons exhibited orexin immunoreactivity. It was concluded that oxytocin, vasopressin and orexin, but not CCK and CRF, immunoreactive hypothalamic neurons may influence both sympathetic and parasympathetic responses of the mandibular gingiva and lower lip. These are common command neurons [64].

We also determined the chemical nature of the first-order sympathetic and parasympathetic neurons innervating the mandibular gingiva and lower lip. The virus-labeled first-order sympathetic neurons in the upper cervical ganglion (SCG) exhibited dopamine β-hydroxylase (DBH) and many of them exhibited neuropeptide-Y (NPY) immunoreactivities. Interestingly vesicular acetylcholine transporter (VAChT) immunoreactive cells were also seen in this sympathetic ganglion but colocalization was not observed with virus labeling. Very few VIP cells were found and there was colocalization between virus labeling and VIP immunoreactivity. In the parasympathetic otic and submandibular ganglia, all virus-labeled neurons exhibited VAChT, some of them showed NPY, DBH or VIP immunoreactivity as well [64].

### 9.2. Autonomic and Sensory Innervation of the Mammary Gland in Lactating Rats

**Figure 1:**
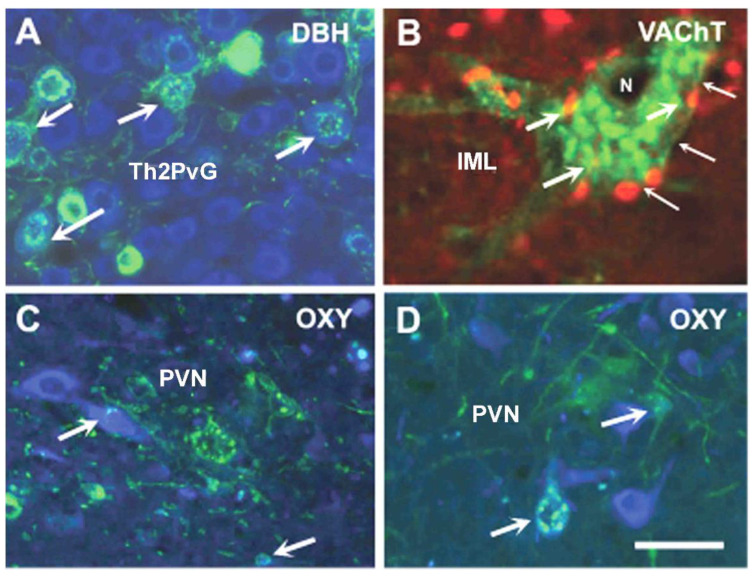
Microphotographs demonstrating GFP-conjugated virus-labeled neurons (green) which also show DBH (**A**, blue color), VAChT (**B**, red color) or OXY (**C**,**D**, blue color) immunoreactivity. (**A**) In Th2PvG, a subpopulation of virus-labeled neurons colocalizes with DBH immunoreactivity. Colocalization is indicated by arrows (blue and green colors in the same cell). (**B**) In a high-power detail of the IML, a virus-labeled neuron (indicated by GFP granules) also contains VAChT-immunopositive granules (red) shown by arrows. Small-headed arrows point out the cell membrane. VAChT granules are usually associated with the inner surface of the membrane. The color of a granule overlapped by the GFP granule is orange. (**C**,**D**). Two details of the PVN show oxytocin-immunopositive cells (blue color) which also contain virus (green color). Where the two colors overlap with each other, the final color is greenish yellow indicated by the arrows. Abbreviations: DBH = dopamine-β-hydroxylase; GFP = green fluorescence protein; IML = intermediolateral cell column; N = nucleus of the cell; OXY = oxytocin; PVN = paraventricular nucleus; Th2PvG = 2nd thoracic ganglion of the paravertebral sympathetic trunk; VAChT = vesicular acetylcholine transporter. Scale = 75 µm in (**A**,**C**,**D**); 25 µm in (**B**). *Reprinted from Acta Physiologica Hungarica 99, 148–158, 2012. Köves K, Györgyi Z, Szabó FK, Boldogkői Zs. Characterization of the autonomic innervation of mammary gland in lactating rats studied by retrograde transsynaptic virus labeling and immunohistochemistry* [65].

We also investigated the innervation of mammary glands in lactating rats [65]. The first and second nipples and the underlying mammary glands were infiltrated with the virus (MemGreenPRV-R). When the animals were sacrificed two days after injection, labeling appeared in the dorsal root ganglia at the level of thoracal (Th) 2-6 segments, but not in the dorsal rootlets and dorsal horn of the spinal cord. The retrograde virus could not label the central axon of the pseudo-unipolar sensory neurons because it could not be transported in an anterograde manner. GFP labeling was also observed in the ipsilateral upper paravertebral sympathetic ganglia and the virus could enter the ipsilateral lateral horn indicating that the virus could be retrogradely transported through a synapse to the next member of the descending neuronal chain. A significant number of labeled cells was seen in the Th2-Th5 segments, and just a few in the Th6 segment. Below this level, there was no labeling in the lateral horn. When the animals were sacrificed three days after the injection, a few labeled neuronal cell bodies appeared in the brain stem and the hypothalamus. When the animals were sacrificed four days later, many labeled cells were seen on both sides in the VLM and scattered cells in other brainstem regions including LC, raphe nuclei and periaqueductal gray matter. The virus-labeled cells were not evenly distributed in both sides of the hypothalamus. Many labeled cells were observed in the PVN on the ipsilateral side and only a few in the contralateral side. Immunostaining revealed that in the paravertebral ganglia, many small DBH immunoreactive and a few large VAChT immunoreactive neurons contained the virus. A subpopulation of GFP-labeled perikarya showed DBH immunoreactivity. The DBH immunoreactive material homogenously filled out the cells; GFP which indicates the presence of virus, formed small green granules (Figure 1A). In the lateral horn, VAChT immunoreactivity was also seen in virus-labeled neurons. VAChT immunoreactivity was located in red granules and in many cases, these granules were associated with the inner surface of the cell membrane. A few VAChT granules were present inside the cytoplasm between the green granules. Green granules represent the presence of the replicated virus (Figure 1B). We did not observe DBH-immunoreactive perikarya in this region, but a very dense DBH-positive fiber network was present. A subpopulation of GFP-labeled neurons in the PVN also exhibited oxytocin immunoreactivity (Figure 1C,D).

### 9.3. Usefulness of Fluorescence Virus Tracers in the Study of Neural Pathways at Different Ages

**Figure 2:**
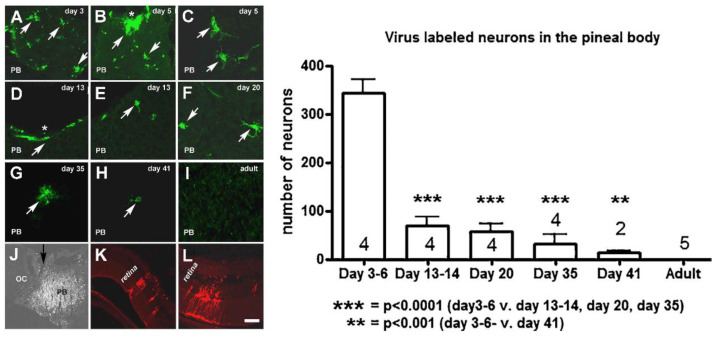
(**A**–**I**) Microphotographs show representative sections from the PB of rats of different ages after intravitreal administration of memGreen-RV. The white arrows indicate virus-labeled perikarya. The asterisk shows the rostral pole where the neuronal fibers enter the organ. (**J**) A black arrow shows the site of administration of Ka-VHSmCherry-A-RV into PB and (**K**,**L**) demonstrates the retinal labeling after this intervention. Abbreviations: OC = occipital cortex; PB = pineal body. Scale = 500 μm in J and 50 μm in A–I and K and L. The chart bar shows the quantitative assessment of the number of virus-labeled cells in the PB of rats with different ages. The number of cells tremendously decreased after 6 days following birth, with a continued gradual decrease until puberty. By puberty, they completely disappeared. In adult rats, neurons were not found in the PB. The numbers in or above the bars indicate the number of animals. *Reprinted from Neuroscience Letters, 665, 189–194, Csáki et al. Ontogenesis of the pinealo-retinal neuronal connection in albino rats. 2018. with permission from Elsevier. ISSN: 0304-3940* [73].

The presence of neuronal cell bodies in the pineal body (PB) is not generally accepted despite the fact that evidence indicates their existence in this organ. Vigh-Teichmann and her co-workers [66], using GABA immunohistochemistry, identified neurons in adult cat PB, mainly in the subependymal layer. Immunonegative neurons were also seen near the posterior and habenular commissures and their axons left the organ representing the “pinealofugal pathway”. In adult rodents (rat, mouse, hamster, guinea pig), researchers did not find neurons, only GABA-positive fibers which may be of central origin and innervate pinealocytes [67]. In other animals, such as cotton rats and monkeys, neurons in the PB were observed [68,69]. The retino–hypothalamo–pineal connection was identified earlier [70,71], but a reverse connection has not yet been revealed. In our experiment, a modified PRV-Ba virus (MemGreen PRV-R), spreading exclusively in the retrograde direction, was successfully applied as a neuronal tracer in adult, neonatal, pre- and post-pubertal animals identifying the neuronal connection between the PB and retina. In the first experiment, we used adult Sprague–Dawley rats and golden hamsters. The virus was injected into the vitreous body of the eye. Centrifugal fibers, present in the retina, picked up the virus and transported it in a retrograde direction through the optic nerve to the central nervous system. The animals were sacrificed 4 days later. The whole PB was sectioned and green fluorescence was looked for using a fluorescent microscope. The transsynaptic GFP expressing the virus did not reach the PB in adult rats; however, in adult hamsters, we observed labeled neurons. The number of neurons was relatively limited, 5–36 in the whole organ [72]. We decided to look for neurons in young rats from neonatal to pre- and post-pubertal ages. The MemGreenPRV-R virus was injected into the vitreous body of the eye. Two to three days later, green fluorescence was looked for in the PB. The labeled cells were counted. Surprisingly, in neonatal rats we identified about 300 cells. The number of cells gradually decreased towards puberty in both male and female rats [73]. Electron microscopic examination verified that the labeled cells were neurons. Immunohistochemistry for neuronal nuclear protein (NeuN) also revealed neurons in the neonatal rat PB [74]. When a virus, spreading in the anterograde direction and expressing red fluorescent protein (KA-GEI-memTomato-A-RV), was injected in the PB of six-day-old rats, two days later it appeared in the retina. It means that the virus reached the retina through a neuronal chain. Figure 2 demonstrates the virus labeling in the rats with different ages and the labeling in the retina after the virus injection into the PB of a six-day-old rat. This experiment highlighted the development of the retino-to-pineal connection in rats [73]. No data are available today on whether the number of pineal neurons decreases similarly from the neonatal to adult age in mammals (other than rats), but it is strongly suggested. No physiological data are found which would fully explain the role of this connection. Because in rats the number of neurons in the PB disappears by puberty, it is possible that they have a role in inhibiting the gonadotropin release from the pituitary gland. We did not investigate the complete step-by-step connection with the retina. It is strongly suggested that there is a connection between the PB and the gonadotropin-inhibiting hormone (GnIH)-producing neurons, which were identified about 20 years ago in the dorsomedial hypothalamic area in mammals [75,76].

**Figure 3:**
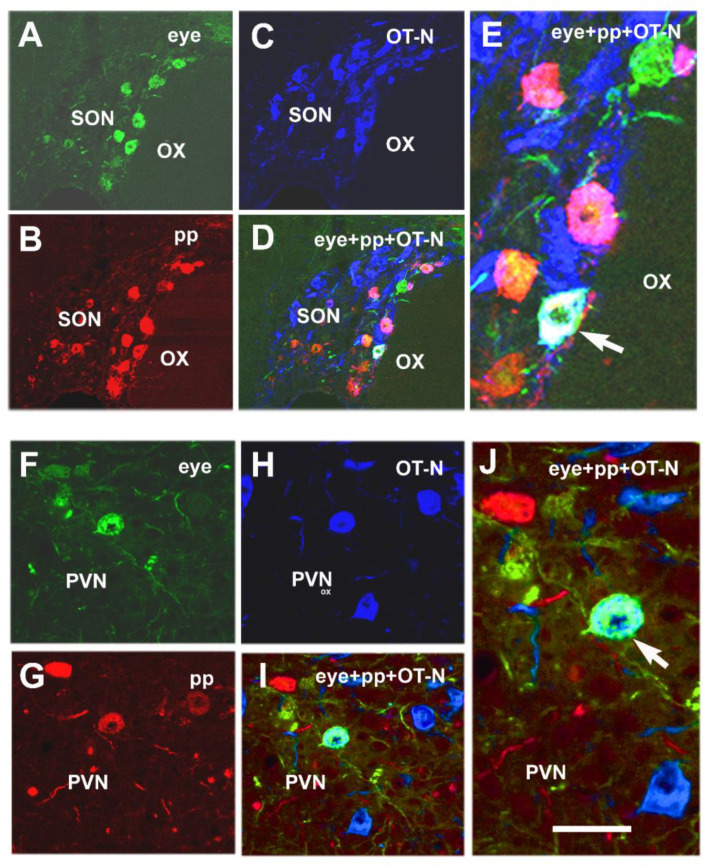
Microphotographs showing oxytocin (OT-N) immunoreactivity in double-virus-labeled neurons in the SON and PVN. (**A**) Ka-gEI-memGFP-RV green labeling in the SON of an intact rat. (**B**) Ka-gEI-memTomato-A-RV red labeling in the SON of the same section of an intact rat. (**C**) OT-N immunostaining in the SON of the same section (seen in blue). (**D**) Triple-labeled neuron in a confocal merge image of the SON (whitish color). (**E**) High-power detail of D. (**F**) Ka-gEI-memGFP-RV green labeling in the PVN of an intact rat. (**G**) Ka-gEI-memTomato-A-RV red labeling in the PVN of the same section. (**H**) OT immunostaining in the PVN of the same section (blue). (**I**) Triple-labeled neuron in a confocal merge image of the PVN (bluish white). (**J**) High-power detail of (**D**). Arrow shows triple-labeled cell in (**E**,**J**). Abbreviations OT-N = oxytocin neurophysin; OX = optic chiasm; PP = posterior pituitary; PVN = paraventricular nucleus; SON = supraoptic nucleus,. Scale: 160 µm in (**A**–**D**) and 40 µm in (**E**), 80 µm in (**F**–**I**) and 40 µm in (**J**). *Reprinted from NeuroSci 2021, 2, 27–44. Csáki* et al. *The Same Magnocellular Neurons Send Axon Collaterals to the Posterior Pituitary and Retina or to the Posterior Pituitary and Autonomic Preganglionic Centers of the Eye in Rats. With permission from MDPI* [77].

In another experiment, we looked for direct neuronal connections between the magnocellular paraventricular and supraoptic nuclei, posterior pituitary and the retina in adult rats. In order to investigate this problem, double-virus labeling and immunohistochemistry were used. The GFP expressing virus (KA-GEI-memGreen-R), spreading retrogradely, was injected in the vitreous body of the eye and a virus, spreading in both ante- and retrograde directions and expressing red fluorescent protein (KA-GEI-memTomato-A-RV), was injected in the posterior pituitary. The axons of the magnocellular neurons could only transport the virus retrogradely toward the hypothalamus because neuronal cell bodies are not present in this organ. Virus labeling appeared in both the supraoptic nucleus (SON) and PVN, and double-virus-labeled cells were also observed. Oxytocin immunostaining revealed that some double-virus-labeled cells contain oxytocin as well. Figure 3 shows triple labeling in the SON and PVN of an adult 2-month-old rat [77].

## 10. Summary and Conclusions

In the past one-hundred years, neuroscience has developed tremendously. One of the most important early discoveries was the theory of contiguity [3]. Decades later, the hypothesis of chemical transmission was born [6]. Its morphological basis, the synapse, was demonstrated by electron microscopy [4,5]. At the same time, the gap junction between nerve elements was discovered [7].

A need arose to explore neuronal circuits. Neuroscientists looked for methods to visualize connections. Among others, tracing techniques seemed suitable. In the beginning, non-transsynaptic tracers, and later, transsynaptic tracers, were introduced in research work. The fluorescent tracers can be visualized easily without other techniques, but it also allows the chemical characterization of labeled neurons by immunohistochemistry. NeuN immunohistochemistry and virus labeling cannot be carried out together because the virus replicates in the nucleus of the neurons and prevents the NeuN staining [74]. Table 1 summarizes neuronal tracers used in the last century.

When we reviewed the development of tracking techniques, it became apparent that there is need for the further development of tracers through human manipulations to adapt them for many other specific purposes.

## Figures and Tables

**Table 1:** Classification of neuronal tracers.

Type of tracers	Direction of Transportation	Visualization	References
Non-fluorescent non-transsynaptic tracers			
Triciated amino acids	Anterograde	Autoradiography	Lasek et al. [9]
WGA (wheat germ agglutinin)	Ante-/retrograde	Avidin-biotinylated HRP (ABC)	Gerfen and Sawchenko [78]
PHA-L (phaseolus vulgaris leucoagglutinin)	Ante-/retrograde	Avidin-biotinylated HRP (ABC)	Gerfen and Sawchenko [78]
CTXB (choleratoxin subunit B)	Retrogade	ABC and alexafluor	Cont et al. [79]
Fluorescent non-transsynaptic tracers			
Evans Blue	Retrograde	Fluorescence microscopy	Kuypers and Huisman [24]
NY (Nuclear Yellow)	Retrograde	Fluorescence microscopy	Bentivoglio et al. [23]
Bp (Bisbenzimide)	Retrograde	Fluorescence microscopy	Bentivoglio et al. [23]
Fb (Fast blue)	Retrograde	Fluorescence microscopy	Kuypers et al. [24]
Tb (True blue)	Retrograde	Fluorescence microscopy	Kuypers et al. [24]
DY (Diamidino Yellow dihydrochloride)	Retrograde	Fluorescence microscopy	Keizer et al. [25]
SITS (Stilbene compound)	Retrograde	Fluorescence microscopy	Schmued and Swanson [26]
SG (Stilbene Gold)	Retrograde	Fluorescence microscopy	Schmued and Fallon [27]
FG (Fluorogold)	Retrograde	Fluorescence microscopy	Schmued and Fallon [27]
Latex microspheres Rhodamine labeled	Retrograde	Fluorescence microscopy	Katz et al. [28]
Tracers passing gap junctions			
Neurobiotin 286Da	Versatile	Avidin-biotinylated HRP (ABC), Streptavidin-Cy3	Vaney [80], Huang et al. [81]
			Mills and Massey [12]
Biotin-X cadaverine 442Da	Versatile	Avidin-biotinylated HRP (ABC), Streptavidin-Cy3	Mills and Massey [12,82]
Lucifer yellow	Versatile	Fluorescence microscopy	Mills and Massey [82]
Non-fluorescent transsynaptic tracers			
Pseudorabies virus (PRV) Ba- DupLac	Ante-/retrograde	Immunostaining hrp or fluorescence microscopy	Boldogkői et al. [34]
Herpes simplex virus (HSV) expressing LacZ	Anterograde	Immunostaining hrp or fluorescence microscopy	Ho and Mocarski [35]
Vesicular stomatitis virus (VSV)	Ante-/retrograde	Fluorescence microscopy	Beier and co-workers [36]
Fluorescent transsynaptic tracers			
KA-GEI-memGFP-RV	Retrograde	Fluorescence microscopy	Boldogkői et al. [47]
KA-GEI-memTomato-A-RV	Ante-/retrograde	Fluorescence microscopy	Csáki et al. [72,74]
VHS-mCherry-A-RV	Ante-/retrograde	Fluorescence microscopy	Csáki et al. [73,74]

## Abbreviations

BDA: biotinylated dextran amine; CCK, cholecystokinin; CRF, corticotropic hormone-releasing factor; DMN, dorsomedial nucleus; DBH, dopamine-β-hydroxylase; EB, Evans Blue; EGFP, enhanced green fluorescence protein; EM, electron microscope; FB, fast blue; FG, FluoroGold; GABA, gamma-amino-butyric acid; GAD, glutamate decarboxylase; GFP, green fluorescent protein; Gig, gigantocellular reticular neurons; GnIH, gonadotropic-inhibiting hormone; GnRH, gonadotropic hormone-releasing hormone; HSV, herpes simplex virus; IML, intermediolateral cell column in the spinal cord; LC, locus ceruleus; NeuN, neuronal nuclear protein; NY, nuclear yellow; PB, pineal body; Pf, perifornical area; PV, parvalbumin; PRV, pseudorabies virus; PVN, paraventricular nucleus; RHT, retinohypothalamic tract; RV, rabies virus; rVLM, rostral ventrolateral medulla; SON, supraoptic nucleus; TB, true blue; VAChT, vesicular acetylcholine transporter; VIP, vasoactive intestinal polypeptide; VSV, vesicular stomatitis virus.
